# OnabotulinumtoxinA injections in chronic migraine, targeted to sites of pericranial myofascial pain: an observational, open label, real-life cohort study

**DOI:** 10.1186/s10194-017-0781-7

**Published:** 2017-07-21

**Authors:** Danièle Ranoux, Gaelle Martiné, Gaëlle Espagne-Dubreuilh, Marlène Amilhaud-Bordier, François Caire, Laurent Magy

**Affiliations:** 1 Department of Neurosurgery, Centre Hospitalier Universitaire de Limoges, Limoges, France; 2 Pain Center, Centre Hospitalier Universitaire de Limoges, Limoges, France; 3 Pain Center, Centre Hospitalier de Guéret, Guéret, France; 4 Department of Neurology, Centre Hospitalier Universitaire de Limoges, Limoges, France

## Abstract

**Background:**

OnabotulinumtoxinA has proven its efficacy in reducing the number of headache days in chronic migraine (CM) patients. The usual paradigm includes 31 pericranial injection sites with low dose (5 U) per site. The aim of this study is to present the results obtained using a simpler injection protocol of onabotulinumtoxinA, with injection sites targeted to pericranial myofascial sites of pain.

**Methods:**

Observational, open label, real-life, cohort study. We enrolled 63 consecutive patients fulfilling the diagnostic criteria of CM, and refractory to conventional treatments. The patients were injected using a “follow-the-pain” pattern into the corrugator and/or temporalis and/or trapezius muscles. The doses per muscle were fixed. According to the number of muscles injected, the total dose could vary from 70 to 150 U per session. Patients were considered responders if they had a ≥ 50% decrease in number of headache days in at least two consecutive injection cycles.

**Results:**

Forty one patients (65.1% in intention to treat analysis) responded to treatment. In 70.7% of responders, the effect size was even higher, with a reduction ≥70% in the number of headache days. The associated cervical pain and muscle tenderness, present in 33 patients, was reduced by ≥50% in 31 patients (94%). Triptan consumption dramatically decreased (81%) in responders. The trapezius was the most frequently injected muscle. We observed no serious adverse event. The mean patient satisfaction rate was 8.5/10.

**Conclusions:**

This study provides additional robust evidence supporting the efficacy of onabotulinumtoxinA injections in CM. Furthermore, the paradigm we used, with reduced number of injection sites targeted to pericranial myofascial sites of pain, may provide evidence in favor of the implication of myofascial trigger points in migraine chronicization.

**Trial Registration:**

ClinicalTrials.gov Protocol Record I17022 ClinicalTrials.gov Identifier: NCT03175263.

Date of registration: June 7, 2017. Retrospectively registered.

## Background

Chronic migraine (CM) is defined as headache occurring on 15 or more days per month for more than 3 months, which has the features of migraine headache on at least 8 days per month [1]. This disabling condition affects approximately 1–2% of the general population [2] and has a much stronger impact on quality of life and employment than episodic migraine [3]. The reason why episodic migraine becomes chronic remains poorly understood. The most recent data highlight the role of decreased activity of the descending pain-modulating network, and of sensitization of central structures including the thalamus, periaqueductal grey matter, and spinal trigeminal ganglion [4]. Some risk factors for chronicization of migraine have been identified, including frequency of migraine attacks, obesity, excessive use of opioids and barbiturates, caffeine overuse, stressful life events, sleeps disorders and cutaneous allodynia [5]. Most patients with CM overuse medication, but it is unclear whether this fact is a cause or a consequence of chronicization of migraine, and in ICHD-3 (International Classification of Headache Disorders 3) beta version the diagnosis of CM can be made regardless of whether the patient overuses medication or not [1].

Treatment of CM is challenging, since triptans or ergot derivatives are inconsistently effective. It requires a multifaceted approach, including lifestyle modifications, management of triggering factors, education, support, and behavioral therapy [4]. Drug withdrawal is considered mandatory by most physicians who believe that acute medication overuse is the major cause of migraine chronicization. Some studies, however, have demonstrated that CM patients’ condition could be improved without drug withdrawal [6]. Furthermore, the modalities of medication discontinuation are still a matter of debate [7]. Pharmacological treatment options are limited, relying on classical oral prophylactic drugs. With the exception of topiramate, however, these agents have not been specifically evaluated in patients with CM. Non-pharmacological options include invasive procedures such as occipital nerve stimulation, even if the first randomized studies did not confirm the promising preliminary data [8]. In 2010, two large, placebo-controlled trials, PREEMPT (Phase III Research Evaluating Migraine Prophylaxis Therapy) 1 and 2, demonstrated that OnabotulinumtoxinA (OnaA) (Botox®, Allergan Inc) significantly decreased the severity and frequency of CM headache [9, 10]. The design of these studies has been criticized [11, 12], stressing several methodological weaknesses such as the change of primary outcome measure between the two studies, and possibly inadequate blinding. Furthermore, the placebo effect was particularly strong in these trials. These results, however, led the Food and Drug Administration to approve Botox® use in CM, and the recent update of the American Academy of Neurology guidelines recommends (level A) the use of OnaA in CM to reduce the number of headache days [13].

The PREEMPT trials injection scheme relies on the observation that OnaA injections applied for hyperfunctional facial lines are able to alleviate migraine symptoms [14], and consists in 31 injection sites throughout pericranial muscles. We postulated that, instead of injecting small doses in multiple sites, it could be more appropriate to inject higher dosage in a limited number of muscles known to be a source of myofascial pain in CM patients, such as the corrugator, temporalis and trapezius muscles [15–20].

We present here the clinical outcome of a cohort of 63 patients treated with this paradigm of injection.

## Methods

### Patients

In France, OnaA has not yet been approved for the treatment of CM. Since 2008, in a compassionate use, we offered these patients to receive OnaA injections on an off-label basis. Patients were eligible to OnaA treatment if they fulfilled International Headache Society (IHS) criteria for CM, regardless of whether they had an excessive abortive drug intake or not. This is in line with the current ICHD3 definition of CM in which medication overuse no longer excludes the diagnosis of CM [1]. The patients were considered refractory if they did not have any response to at least two prophylactic antimigrainous agents. They had to sign informed consent to receive OnaA treatment, and the off-label status of this drug in France was stressed. Patients were initially evaluated and followed-up by one of the authors or were alternatively referred by neurologists or pain specialists from elsewhere.

### Study design (Figure 1)

This study was an observational, open-label, cohort-study conducted in accordance with the principles of the Helsinki Declaration. We prospectively and systematically recorded data from the patients and analyzed them retrospectively. During a first phase, called *adaptation period*, the injector (DR) used a follow-the-pain approach in order to determine the optimal injection scheme for each individual. The possible injection sites were the corrugator, temporalis, and trapezius muscles. Patients were systematically asked about the usual topography and time course of migraine attacks, and the existence of pain or stiffness of the cervical muscles. Examination searched for muscle tenderness and the existence of myofascial trigger points (TrPs). The decision to inject a single muscle relied on data from questioning and examination. The existence of referred pain patterns characteristic of TrPs on questioning as well as the identification of TrPs on examination were arguments to inject a given muscle. As an atlas of muscle referred pain, we used the manual by Travell and Simons [21]. For example, the existence of pain in any or all of the upper teeth during most migraine attacks in an individual was considered suggestive of myofascial involvement of the temporalis muscle. If the pain was predominantly located in, or started from the frontotemporal area, or if TrPs were demonstrated in corrugator and temporalis muscles, or if the topography of pain was suggestive of myofascial involvement, both muscles were injected bilaterally. When the patients had predominant pain in the back of the head, or when their headache pain frequently started and/or ended in the trapezius muscles, both trapezius muscles were injected. All muscle groups were injected if pain was both frontotemporal and cervico-occipital. When this first set of injections was efficacious, patients were re-injected in the same manner at the time when the frequency of headache days definitely increased. In the absence of efficacy, the paradigm was modified using the same follow-the-pain approach. Once the best procedure was determined for each patient, it was reproduced at each subsequent injection session. This adaptation phase could necessitate up to three sessions. The *observation period* started 8 weeks before the first efficacious injection and ended 2 months after the second consecutive efficacious injection, or in case of inefficacy. Throughout the adaptation and the observation phases, patients kept a headache diary where they were asked to note the days with headache and the use of rescue medication. The use of prophylactic drugs was allowed, but the dosage was maintained during the two phases. The *extension period* included all treatment cycles after the observation phase. During this period, the injector was allowed to modify the dosage and sites of injections using an even more tailored, follow-the pain approach.

**Fig. 1:**
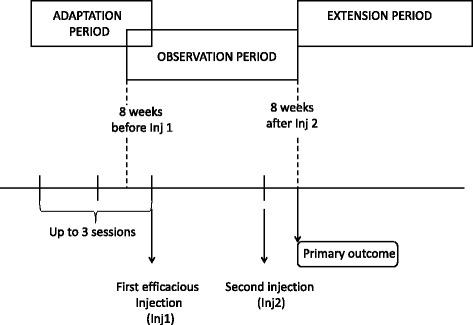
Flow-chart. During a first phase, called *adaptation period*, we used a follow-the-pain approach in order to determine the optimal injection scheme for each individual. Once the best procedure was determined for each patient, it was reproduced at each subsequent injection session. This adaptation phase could necessitate up to three sessions. The *observation period* started 8 weeks before the first efficacious injection and ended 2 months after the second consecutive efficacious injection, or in case of inefficacy. Throughout the adaptation and the observation phases, patients kept a headache diary where they were asked to note the days with headache and the use of rescue medication. The *extension period* included all treatment cycles after the observation phase. During this period, the injector was allowed to modify the dosage and sites of injections

### OnaA injections: Dosage, dilution

The doses per muscle, as well as the injection technique, were predetermined according to our previous experience in CM (DR, unpublished data), and were off-label as well. The corrugator muscle is located in the supratrochlear region. The patient was asked to furrow his/her brow to activate the muscle, allowing an easy insertion of the needle. In order to inject the temporalis muscle, the patient was invited to chew gum, allowing to identify the most active part of the temporalis muscle. The patient was then asked to stop chewing, keeping the teeth clenched. The needle was inserted into the muscle, the extremity of the needle being directed backwards. Then, the patient was invited to relax and the dose was administered into that point. For the trapezius muscle, the injections were also protocol-driven. The dose was distributed into 3 equally-distant sites along the lower part of this muscle. So, we targeted sites of myofascial pain, which are thought to contain TrPs [22] but did not make any effort to target TrPs themselves as sites of injection.

Variation of the dosage was not allowed during the observation phase. The doses of OnaA administered to the corrugator, temporalis and trapezius muscles were respectively 5 U, 30 U, and 40 U. Thus, the overall administered dose could vary from 70 U by session if only the facial muscles were injected to 150 U if all muscle groups were injected. The 100 U vial of Botox® was reconstituted with 2 ml of saline for cervical muscles and with 1 ml for facial muscles. The choice of employing an increased concentration for the corrugator and temporalis muscles aimed to limit unwanted diffusion of the toxin to the adjacent muscles and the subsequent side effects such as ptosis. It has been demonstrated that the injected volume is a major factor affecting the diffusion of botulinum toxin, independently from the dose. In other words, the more concentrated the toxin, the more limited the spreading of botulinum toxin [23]. Additionally, when the total dose is distributed in aliquots of smaller doses along the muscle, the diffusion of the product increases inside the muscle [23]. That is why we used a higher dilution for the trapezius muscles, as well as a multipoint injection procedure, in order to increase the diffusion of the toxin within such a large muscle.

### Outcome measures

In accordance with current guidelines of clinical trials in CM [24], the primary outcome measure was mean change from baseline in frequency of headache days (as recorded in the patient diary) for the 2 months-period ending with week 8. Baseline was defined as the 2 months-period before the first efficient injection. The patients were considered responders if they had a ≥ 50% decrease in headache day frequency in at least two consecutive sets of injections.

The secondary outcome measures were the proportion of patients with a ≥ 70% decrease in headache day frequency, the decrease in triptan consumption, the time to efficacy onset, the duration of therapeutic effect (this was possible because retreatment was administered only when the patients needed to be reinjected ie at the time when the frequency of headache days definitely increased), and the assessment of patient satisfaction on a 0 to10 numerical scale (0 = no improvement, and 10 = maximum possible improvement). Data from the follow-up beyond the 2 first efficient injections were recorded.

### Statistical analysis

Fisher exact test was used to compare frequencies between groups. Student t test was used to compare the means of two samples. A *p* value <0.05 was considered significant.

## Results

Sixty three consecutive patients were referred to our center for refractory CM from 2008 to 2015. All screened patients consented to receive OnaA injections and signed informed consent. They were 43 females and 14 males, aged 17 to 85 years (mean: 44.3).

The results are summarized in Tables 1 and 2. Five patients dropped out early after the first injection session due to inefficacy (*n* = 2), living far from the hospital (*n* = 1), other health problems (*n* = 1), or personal reasons (*n* = 1). One other patient, aged 85 years, was excluded because of cognitive troubles making the assessment of response to OnaA difficult. Those 6 patients were included in the intention-to-treat (ITT) analysis. Sixteen patients did not respond. Forty one patients (65.1% in the ITT analysis, 72% in the per-protocol analysis) reached the primary efficacy endpoint (a ≥ 50% decrease in headache day frequency in at least two consecutive injection cycles). In 29 out of these 41 patients (70.7%), the reduction in headache days was ≥70%, with 9 patients (22% of responders) virtually headache-free (≤ 1 headache day per month.

**Table 1:** Primary outcome measure

	Dropped out	Non responders	Responders	Proportion of responders	
				ITT analysis	Per-protocol analysis
N	6	16	41	65.1%	72%

*ITT* Intention To Treat

**Table 2:** Secondary outcome measures

	Number	Percent of responders
Patients with ≥70% reduction in headache day frequency	29	70.7%
Patients with ≤1 headache day/ month	9	22%
Patients with ≥50% reduction in intercritic cervicalgia	31	94% ^a^
	Mean	
Percentage of reduction in triptans consumption vs baseline	81% ^b^	
Patient satisfaction mean on a numerical scale from 0 to 10 (min-max)	8,6 (6.5–10)	

^a^Percentage of the 33 patients with cervicalgia at baseline

^b^Percentage of the 28 patients who took triptans at baseline

Cervical pain and muscle tenderness were particularly frequent at baseline, present in 33 responders, and was reduced by ≥50% in 31 of them (94%) after treatment.

The optimal treatment regimen, determined during the adaptation period, included injections into the corrugator, temporalis and trapezius muscles bilaterally (total dose: 150 U) in the majority of patients (33/41, 80.5%). In five patients the treatment was administered into both trapezius muscles only (total dose: 80 U). The last three patients were injected into the corrugator and temporalis muscles only (total dose: 70 U). The trapezius muscle appeared to be a key target for OnaA injections in CM, since 38 patients out of 41 (92.7%) required injections into this muscle to improve.

At baseline, 15 patients did not take any triptans because of contraindication, loss of efficacy, or because triptans had never been effective. In responders, among the 28 triptan consumers, rescue drug consumption dramatically decreased (mean: 81%). Most patients reported a much better efficacy of the triptans on residual migraine attacks compared to the pre-treatment period.

There was no statistically significant difference between the responder and non-responder groups in terms of age, gender, mean baseline number of headache days, consumption of triptans at baseline, or the presence of prodromal, percritic or intercritic cervical muscle pain and tension (Table 3). By contrast, the presence of the three characteristics in the same patient (combination of a painful tension of the cervical muscles between, preceding and accompanying the attacks) was significantly more frequent in the responder group compared to the non responder group (*p* = 0.002).

**Table 3:** Description of the population and comparison of data in responders vs non responders

	All	Responders	Non responders	Comparison
	(*N* = 57)	(*N* = 41)	(*N* = 16)	
Mean age (min-max)	44.3 (17–72)	43.2 (17–72)	48.4 (22–64)	NS
Female/Male	43/14	32/9	11/5	NS
Migraine with aura/without aura	7/50	6/35	1/15	NS
No triptans use	15	13	2	NS
Medication overusers	33	19	14	NS
Mean baseline number of headache days per month	23.12	22.63	24.36	NS
Baseline consumption of triptans/month	17.8	15.76	20.66	NS
Intercritic cervicalgia	42 (73.7%)	33	9	NS
Percritic cervicalgia	45	35	10	NS
Prodromal cervicalgia	35	30	5	NS
Combination of intercritic, percritic and prodromal cervicalgia	30	27	3	*P* = 0.002

*NS* non-significant

Surprisingly, the onset of efficacy was abrupt in most patients, the maximum of benefit being reached in a few days, after a latency ranging from 5 to 30 days (mean = 14.8 days). The duration of action ranged from 3 to 4 months.

The injections were well tolerated. The only significant adverse event we observed was local myalgia when the trapezius muscle was injected. This pain occurred 1 to 5 days after injection, and could last up to 15 days. It was qualified as severe in 4 patients (9.7%) and, interestingly, did not recur, or recurred very moderately, during the subsequent injection cycles. We observed no eyelid ptosis, perhaps due to the high concentration (1 ml/100 OnaA Units) used in our study to inject facial muscles. The patient satisfaction was particularly high, with a mean score of 8.6 (6.5–10) on a 0–10 numerical scale.

### Extension phase

Two of the 16 patients considered to be non-responders claimed receiving retreatment because their migraine headaches were shorter-lasting, less severe, and easier to treat. Among the 41 responders, 34 are still under treatment, with a mean number of injection cycles of 5.95 (2–18). In those patients, the clinical benefit was maintained between each injection session and lasted three to 4 months. Seven patients discontinued treatment: 2 because they had achieved a sustained clinical benefit and the others due to personal reasons or lack of compliance. In this phase, patients were not required to keep a headache diary, but there was a trend towards a lengthening of intercycle intervals, and an increase in size effect with time. According to the patient pain pattern, additional muscles were injected in some patients, such as procerus, splenius capitis, suboccipitalis and masseter muscles.

## Discussion

Myofascial pain syndromes have been described in CM patients in various pericranial sites including the neck muscles, the supratrochlear and corrugator region, and the temporalis muscles [15–20]. This preliminary study shows that OnaA injections targeted to those sites provide a high rate of response (65.1%, ITT) in CM. Furthermore, the magnitude of the response was large, with 70.7% of the responders exhibiting a ≥ 70% decrease in headache day frequency in at least two consecutive sessions of injections, and 22% being virtually headache-free. Finally, we observed a dramatic decrease (81%) in triptan consumption in responders.

Most patients had not experienced an improvement of this magnitude in years, as attested by the high degree of patient satisfaction (mean 8.6/10).

Our study, as well as the other recently published real-world experiments addressing the same issue [25–31], has some limitations, including the small sample size and the absence of a placebo arm. Placebo effect is particularly high in headache conditions, especially when the treatment is administered by injection. In order to lower the impact of placebo effect on our results, we considered patients as responders only if OnaA treatment was efficacious in two consecutive sets of injections. In addition, some features in the response can hardly be explained only by the placebo effect, such as (i) the reproducibility of results at each injection session (up to 18 per patient); (ii) the fact that the response to treatment in single patients differed according to the muscles injected (for example, some patients did not respond to frontotemporal injections in the adaptation phase, but responded to trapezius muscles injection). Finally, the long latency to efficacy onset we observed (up to 30 days) is unusual for a placebo effect.

Previous real-life studies provided conflicting results, with a ≥ 50% reduction in number of headache days achieved in a range as wide as 17.4 to 63% of patients [25–31]. With a figure of 65.1%, our results are in the higher part of this range, which may be due to the technical choice we have made. Indeed, our plan of injection differed from the PREEMPT protocol they used. We injected only the corrugator muscle on the forehead, not the frontalis or the procerus muscles. The temporalis muscle was injected in one site rather than in four, and the only cervical muscle injected was the trapezius muscle, with a higher dose (40 U versus 15 U). This resulted in a simplified paradigm with a maximum of 10 injections sites. Since the pivotal PREEMPT studies, a variety of injection techniques have been proposed, with modification of doses or sites of injections. Negro et al. [32] have demonstrated a dose-depending effect of OnaA in patients with CM and medication overuse headache, with a superior efficacy of OnaA 195 U compared to 155 U. Our results suggest that parameters others than the global injected dosage may be of relevance, such as the selection of a limited number of muscles using an individualized follow-the-pain approach, as well as an adequate dosage per muscle. Only a controlled, randomized study would be able to compare the efficacy of PREEMPT paradigm to such a tailored protocol, targeted to sites of myofascial pain. We suggest, however, that the paradigm we used may constitute a promising way to improve the outcome of CM patients treated with OnaA.

This injection paradigm was elaborated with reference to the theory which assumes that the muscle sites with myofascial pain act as triggers to initiate or perpetuate migraine. In support of this hypothesis, it has been demonstrated that inactivation of cervical trigger points by anesthetic infiltrations or manual therapy resulted in reduced migraine number and intensity [18, 19, 32–34]. The present study is to our knowledge the first to use OnaA injections targeted to pericranial muscle pain in CM patients. However, in a 2011 review paper, Gerwin mentioned his personal unpublished experience using a similar approach. He reports that he injects OnaA into the TrPs in the head, neck, and shoulder muscles identified by physical examination, and finds that 50% of patients treated in this way are headache-free, with an additional 30% significantly improved [35]. Both approaches are very close together. The only difference is that we did not target TrPs as the sites of injection. Identification of TrPs within a given muscle was just for us a mean to correctly select the muscles to inject. In a key paper about myofascial headache, Fernandez-de-las-penas [22] suggests the crucial role of TrPs in generating muscle pain. Indeed, evidence supports that active TrPs release algogenic substances that are susceptible to promote the sensitization of muscle nociceptive nerve terminals, which may be responsible for muscle pain. In turn, the sensitized nerve ending liberate vasoactive neuropeptides such as calcitonine gene-related peptide (CGRP), Substance P and Glutamate, leading to a local neurogenic inflammation [22]. Since it is currently established that OnaA inhibits exocytosis of acetylcholine as well as multiple neurotransmitters including serotonin, dopamine, noradrenaline, gammaaminobutyric acid (GABA), enkephalin, glycine, substance P, ATP and calcitonin gene-related peptide (CGRP) [36], we can assume that OnaA may act in CM through a reduction of the peripheral sensitization within the injected muscles. It is however unlikely that the action of OnaA is limited to a peripheral effect. Indeed, the fact that OnaA induces a reduction of migraine attacks frequency implies that a central action also exists in one way or another. Some studies have suggested that myofascial inputs may activate the trigeminovascular system and therefore trigger migraine attacks in migraine sufferers [18]. It can therefore be suggested that an indirect central effect may result from the reduction of nociceptive myofascial input towards central neurons. In addition, we cannot rule out a direct central effect through the retrograde transport of OnaA, which has been demonstrated in numerous preclinical studies [37]. It is unclear, however, whether OnaA axonal transport has a clinical relevance in humans.

Cervicalgia present between migraine attacks was a major concern in our patients, present in 73.7% of cases. This is in keeping with studies that found a higher prevalence of neck pain disorders in patients with chronic rather than episodic migraine [38]. The significance of the cervical muscle tenderness observed in chronic headache is not fully understood, but is thought to result from myofascial pain [22]. We found that the combination of prodromal, percritic and intercritic cervicalgia was a predictive factor of response to OnaA treatment in CM patients, suggesting that the more severe the cervical myofascial disorder, the better the outcome. We also found that 94% of patients with neck muscle tenderness had a ≥ 50% reduction in cervicalgia intensity. Both findings are in line with our assumption that OnaA acts in CM at least partly by relieving the myofascial component of pain. We also showed that most patients reported a better efficacy of triptans after OnaA treatment. We think this supports the view that OnaA acts on myofascial pain, which is by definition unresponsive to triptan. Once relieved, the remaining pain is purely migrainous and is therefore triptan-responsive.

We found that the latency of therapeutic effect was long-lasting, with a mean of 14.8 days (up to 30 days). This finding was unexpected, since the delay of action of OnaA is estimated around a few days in the classical indications of OnaA such as dystonia and spasticity [39]. To our knowledge, only one other study addressed this issue [28]. The authors found that the first signs of therapeutic effect started after a mean of 5.5 days, which suggests a progressive onset of improvement. The pattern of response in our patients was quite different. Patients reported a delayed, but rapidly occurring improvement. This difference may be due to the injection paradigm we used, targeted to sites of myofascial pain. If so, the long delay to onset we observed could be an argument supporting a central participation in the mechanism of action of OnaA in CM.

Our results also raise the concern of the role of drug withdrawal in the management of CM. In the present study, we found that OnaA treatment itself led to a dramatic reduction of migraine rescue medication intake (81%). Thus, we propose that, in CM patients, (i) drug abuse may be a consequence of the ancillary myofascial pain rather than the cause of migraine chronicization, and (ii) OnaA treatment should be discussed before considering medication withdrawal.

## Conclusions

We conclude that specifically targeting myofascial pain sites with selective OnaA injections may be a safe and effective option in CM treatment. Further larger, placebo-controlled studies are needed to compare the present protocol with the fixed “multipoint-low dose per point” PREEMPT protocol. If our results were confirmed by further studies, it could be suggested that myofascial pain and TrPs may contribute to headache pain in CM patients and constitute an important factor of migraine chronicization.
